# Women’s Autonomy and Intimate Partner Violence in Peru: Analysis of a National Health Survey

**DOI:** 10.3390/ijerph192114373

**Published:** 2022-11-03

**Authors:** Francisco A. Barón-Lozada, Gianfranco W. Basualdo-Meléndez, Rodrigo Vargas-Fernández, Akram Hernández-Vásquez, Guido Bendezu-Quispe

**Affiliations:** 1 Facultad de Ciencias de la Salud, Universidad Científica del Sur, Lima 15067, Peru; 2 Centro de Excelencia en Investigaciones Económicas y Sociales en Salud, Vicerrectorado de Investigación, Universidad San Ignacio de Loyola, Lima 15024, Peru; 3 Centro de Investigación Epidemiológica en Salud Global, Universidad Privada Norbert Wiener, Lima 15046, Peru

**Keywords:** domestic violence, personal autonomy, health surveys, Peru

## Abstract

To assess the association between women’s autonomy and intimate partner violence (IPV) against women of childbearing age. Secondary analysis of the 2019 Demographic and Family Health Survey (ENDES-acronym in Spanish) was carried out. The study population was women aged 15–49 years who are currently married or living with a partner. A Poisson family generalized linear regression model was estimated to calculate adjusted prevalence ratios (aPR) for the association between women’s autonomy and IPV with their respective 95% confidence intervals (CI). Data from 18,621 women were analyzed. The highest proportion of women had low autonomy (low: 42%; moderate: 39.2%; high: 18.8%). A prevalence of IPV of 40.1% was found (psychological/verbal: 38.8%; physical: 8.8%; sexual: 2.3%). The adjusted model found that women with a low level of autonomy (aPR: 1.15, 95%CI: 1.01–1.31) had a higher prevalence of IPV compared to women with high autonomy. This association was also found for the specific case of psychological/verbal violence (aPR: 1.15, 95%CI: 1.01–1.31). No association was found between women’s level of autonomy and physical or sexual violence by a partner. Four out of 10 women of childbearing age have experienced IPV in the last 12 months. In general, women with lower levels of autonomy are more likely to present IPV compared to women with high autonomy.

## 1. Introduction

Intimate partner violence (IPV) is a public health problem that has negative consequences on women’s health and violates human rights [1]. The type and nature of the violent acts vary, ranging from psychological, physical, sexual, and controlling behaviors inflicted on women and occurring within an environment of marriage or cohabitation with a partner [1]. Although the United Nations Sustainable Development Goal 5.2 seeks to eliminate all forms of violence against women and girls in public and private spheres [2], it is estimated that more than 10% of women aged 15–49 years suffered physical and/or sexual violence by an intimate partner in 2018 [3]. Likewise, it is estimated that IPV generated more than eight million disability-adjusted life years (DALYs) due to mental health disorders and the human immunodeficiency virus (HIV); more than 4 million years of life lost (YLL), and years lost due to disability (YLD), and more than 80 thousand deaths in 2019, being the nineteenth leading cause of death in the world [4]. However, low and middle-income countries could be more affected by the consequences of IPV because of the higher prevalence of this type of violence in these countries compared to high-income countries [3].

In Latin America and the Caribbean (LAC), it is estimated that between 25.5% and 46.4% of women have experienced IPV in the last 12 months [5,6]. LAC lifetime prevalence of IPV is one of the highest in the world (29.8%), after other regions such as Africa (36.6%), Eastern Mediterranean (37.0%) or Asia (37.7%) [7]. Peru is one of the countries with the highest prevalence of IPV in the LAC region, estimating that 57.7% of married or cohabiting women have experienced IPV at some point in their lives in 2019 [8]. In LAC, women possess sociodemographic and cultural characteristics that increase the likelihood of experiencing attitudes of violence by their partner, including a greater tolerance to violence due to an upbringing that generates submissive and dependent behavior towards their spouses and preferences centered on the home and family. In addition, many often consider that if they do not act this way, they may suffer violent acts by their partner [9,10]. Likewise, socioeconomic disadvantages and a low educational level compared to their partner generate a greater attitude of submission and male dominance [10]. Moreover, cultural aspects place men in a dominant role over women and idealize masculine behavior that is associated with aggressiveness, power, and strength, with IPV being a way of demonstrating their authority [10,11]. However, capabilities such as autonomy that allow women to make decisions freely about various aspects of their lives would help to reduce attitudes towards violence by their partners [12].

Biomedical literature describes women’s autonomy as a factor associated with the presence of IPV [13,14]. Regarding the operational definition of autonomy, several studies have described differences in its composition and methods of measurement. However, there are common components that would help to delimit the necessary aspects of women to be autonomous, such as their participation in economic decision-making in the home, free transit, and health, as well as negative attitudes towards violence [13,15,16]. While the terms autonomy and women’s empowerment may be interchangeable, there are differences between their definitions [17]. On the one hand, autonomy refers to the ability to make decisions and exercise control over one’s own economic, material and social resources or in collaboration with one’s spouse or partner, while empowerment is characterized by the ability to resist controls over one’s own life and the denial of one’s rights [17]. Demographic studies conducted in African countries, in which there is a high prevalence of IPV, describe how low autonomy in economic decision-making increases the risk of all types of violence (physical, psychological and sexual), while at the community level, sexual autonomy was seen to be a positive factor in preventing physical and psychological violence [13,14]. In this sense, women’s exercise of autonomy is a necessary intervention that could promote women’s health and other sociocultural aspects.

Despite the high prevalence of IPV and the structural factors that predispose women to this social problem in LAC, there is little evidence of the association between women’s autonomy and IPV in Peru. Therefore, the objective of this study was to determine the association between Peruvian women’s autonomy and IPV using a nationally representative database to provide an overview of this association in the Peruvian territory.

## 2. Materials and Methods

### 2.1. Study Design and Data Sources

An observational, cross-sectional, and analytical study was conducted using the 2019 Demographic and Family Health Survey (ENDES-acronym in Spanish) database. The ENDES is a nationally representative survey, by urban/rural area and of the 25 departments of Peru, which is conducted by the National Institute of Statistics and Informatics of Peru (INEI-acronym in Spanish) [18]. This survey collects information annually on sociodemographic indicators of the population and is divided into three questionnaires: household (in which the characteristics of the household, as well as its assets, are observed), individual (in which information is collected related to the sociodemographic and economic characteristics of women, fertility, childbirth, sexually transmitted diseases, IPV in women aged 12 to 49 years and characteristics of children under five years) and health (information on the sociodemographic and health characteristics of people aged 15 years and older) [18].

The ENDES sampling is two-stage, probabilistic, stratified, and independent at the departmental level and by urban/rural area. The primary sampling unit of the ENDES is composed of clusters selected by probability proportional to their size [18]. The secondary sampling unit is composed of dwellings selected by balanced sampling using the variables children under five years of age and women of childbearing age [18]. In ENDES, the method used to obtain survey information is the direct interview carried out by duly trained personnel to collect this information during a visit to the selected dwellings [18]. Other methodological details of the ENDES can be consulted in the datasheet [18].

### 2.2. Population

The present study included Peruvian women of childbearing age between 15 and 49 years who are currently with a partner (married or cohabiting) with complete data and who were selected and interviewed using the family violence module of the ENDES 2019 women’s questionnaire.

### 2.3. Dependent Variable

IPV was considered if the woman presented any of the following types of violence exercised by the partner in the last 12 months: (1) verbal or psychological violence is a dichotomous variable with values of yes/no, where yes indicates that the woman has lived/experienced at least one of the following situations: jealousy by the husband, accusations of being unfaithful, impediment of having friendships, limitation of visits or contact with family members, control by knowing where she goes, distrust of the money she handles, things are said or done to humiliate her in front of other people, threats to harm her or someone close to her, the partner threatens to leave the house and take her children; (2) physical violence is a dichotomous variable with values of yes/no, where yes indicates that the woman has lived/experienced at least one of the following situations: pushing, shaking, throwing of objects, slapping or arm twisting, hitting with a fist or some object, kicking or dragging, strangling or burning, threats with knives or a gun; (3) sexual violence is a dichotomous variable with values of yes/no, where yes indicates that the woman has lived/experienced any of the situations mentioned: forced her to have sexual relations or perform sexual acts without her consent; and (4) if the woman has experienced any of the types of violence mentioned above (verbal or psychological, physical or sexual).

### 2.4. Independent Variable

The independent variable was the index of women’s level of autonomy. The selection of women’s characteristics that compose this variable was made based on previous studies [13,19,20,21,22,23] (Table 1). These characteristics are related to four dimensions of women’s lives: decision-making exercised by women in their economy, health, and free movement (visiting relatives); attitude towards violence; socioeconomic aspects of women (employment status in the last 12 months and head of household); and socio-cultural aspects (women’s education, access to radio, television, and newspaper). The coding of each of the women’s characteristics that make up this variable was based on the methodology used in previous studies [13,19,20,21,22,23]. To determine the levels of women’s autonomy, an index was constructed with the sum of the final scores for each of the women’s characteristics, where higher scores indicate greater autonomy. This index of women’s autonomy was categorized into tertiles to delineate its three levels: high, moderate, and low [24].

### 2.5. Covariates

The inclusion of these variables was based on the biomedical literature using variables previously described as related to the variables of interest in the study [13,19,20,21,22,23,25,26,27]. Variables specific to the woman, partner or husband, marriage, and household were considered. Regarding women’s variables, age (categorized into age groups), educational level (no level or primary, secondary, higher), ethnic self-identification (native, non-native), and contraceptive use (yes, no) were included. As for the spouse or partner variables, the educational level of the spouse or partner (no level or primary, secondary, higher) and alcohol consumption (yes, no) were included. Also considered were marriage characteristics such as duration of marriage (0–9, 10–19, 20 or more) and the number of children (0,1–3, 4–7), and household characteristics such as place of residence (urban, rural), wealth quintile (Q1 [poorest], Q2, Q3, Q4, Q5 [richest]) and natural region (Coast, Highlands, Jungle).

### 2.6. Statistical Analysis

All statistical analyses were performed using Stata v.14.2 software (Stata Corporation, College Station, Texas, USA). The ENDES sampling characteristics, including strata weights, weighting factor, and design, were specified using the “svy” command. Characterization of the study population was performed by univariate analyses to report simple frequencies and weighted relative frequencies. Differences between proportions were evaluated using the chi-square test.

To identify the association between women’s autonomy and IPV, generalized linear models of the Poisson family and log link function were estimated. In the first stage, crude prevalence ratios (PR) were estimated with their respective 95% confidence intervals (CI). Subsequently, a multivariate model was estimated to obtain adjusted prevalence ratios (aPR) together with their 95% CI, adjusted for variables that obtained a *p*-value < 0.05 in the bivariate regression models. A *p* < 0.05 was considered statistically significant.

### 2.7. Ethical Considerations

The Institutional Research Ethics Committee of the Universidad Científica del Sur (registration code: 497-2021-PRE15) approved the execution of this study than will serve as a partial requirement for two of the authors to obtain their medical degree (Francisco A. Barón-Lozada and Gianfranco W. Basualdo-Meléndez). ENDES participants gave their informed consent before participating in the survey [28].

## 3. Results

A total of 18,621 women of childbearing age were analyzed. Half were between 15 and 24 years old (50.3%), only 6.6% were of native ethnicity, and 43.2% had a secondary level of education. Regarding the place of residence, more than two-thirds (76.9%) of the women lived in an urban area. With respect to the geographical area of origin, the majority resided in the Coastal region (59.1%). Other characteristics of the women included in the study are shown in Table 2.

The prevalence of IPV was 40.1%. Specifically, 38.8% had experienced psychological/verbal violence, 8.8% physical violence, and 2.3% sexual violence (Table 3). Other characteristics of the women included in the study according to types of violence are shown in Table 3.

In relation to women’s autonomy, a low autonomy (42.0%; 95% CI: 40.6–43.4) followed by moderate autonomy (39.2%; 95% CI: 37.8–40.6) were more frequent (Table 4). There were differences with respect to the women’s levels of autonomy according to characteristics such as age group, ethnicity, educational level, duration of the marriage, number of children, educational level of the couple, alcohol consumption by the couple, place of residence, wealth quintile and natural region of origin. Women with high autonomy were more frequently found in the younger age groups, and in women of non-native ethnicity, with a higher educational level, in more recent marriages, without children, with a partner with a higher educational level, residing in urban areas and the coastal region.

Regarding the association between women’s autonomy and IPV, it was found that women with low autonomy (aPR = 1.15; 95% CI: 1.01–1.31) had a higher proportion of total IPV compared to women with high autonomy (adjusted for woman’s age, woman’s ethnic self-identification, current contraceptive use, relationship duration, number of children, partner’s age, partner’s education level, partner’s alcohol intake, wealth quintile, place of residence and natural region of residence). According to the specific type of violence, psychological/verbal violence was more frequent among women with low autonomy (aPR = 1.15; 95% CI: 1.01–1.31) than those with high autonomy. There was no association between the level of autonomy of women and the types of physical and sexual violence experienced (Table 5).

## 4. Discussion

This study aimed to evaluate the association between the level of autonomy of women and IPV. Low autonomy in Peruvian women of childbearing age was found to be related to psychological/verbal violence, but not to sexual or physical violence.

Nearly half of Peruvian women of childbearing age had experienced at least one episode of IPV at some point in the year prior to the survey, indicating that IPV against women is a highly prevalent problem in the Peruvian population. These data are directly influenced by psychological/verbal violence, as shown in the results of the study with a higher proportion of this type of violence. This type of IPV has been described as the most prevalent in high-income countries such as the United States and the European Union [29] as well as medium and low-income economies, including the countries of the Latin American region [30,31]. In Peru, violence in couple relationships is a reflection of the power relations established by the gender system, which enables intimidation and exercise of control by men over women for the preservation of the existing gender system. [32]. Due to the magnitude of the problem, in Peru the care of women has been promoted with the aim of prevention of IPV. In 2021, the Ministry of Women and Vulnerable Populations of Peru approved the national strategy of “Women free of violence” for the prevention of gender-based violence against women. This strategy aims to be a public management tool that allows articulating projects, programs, and policies of the different sectors and levels of government to guarantee that women can exercise their rights free of violence in the public or private sphere [33].

Worldwide, the prevalence of physical and/or sexual IPV in women of childbearing age who have had a partner at some point is estimated at 27% [3]. The results of the present study show that the prevalence of physical and sexual IPV in Peru is lower than the world average for these types of IPV and that it is also lower than the estimate for other South American countries (25%) [3]. Men exercise physical or sexual violence against a woman because they consider that they have the right to do so since they are considered socially superior and can physically discipline a woman for behavior considered incorrect and physical violence is an acceptable way to resolve conflict in a relationship [34,35]. For this reason, although sexual and/or physical violence are not the predominant types of IPV in Peru, programs of sexual education and prevention of sexual violence against women inside and outside a couple are necessary and must be oriented towards people regardless of gender.

In the study of the relationship between the level of autonomy of women and IPV, it was found that a woman with a low level of autonomy had a higher probability of having suffered IPV. Studies in countries such as Ghana, Zimbabwe, and Pakistan have reported a similar association [13,14,36]. However, this association was not found in other countries such as Malawi, which described no relationship between IPV and women’s autonomy [37]. Cultural differences could explain the discrepancy in the findings between countries, and thus, the problem of IPV should be studied according to the sociocultural context of each country. A previous study on the Peruvian population between 2005 and 2012 identified that women who had greater participation in decision-making at home had a lower probability of presenting IPV [38]. Likewise, an association was specifically found between the level of autonomy of women and psychological/verbal IPV, consistent with reports in the literature regarding some low- and middle-income countries [13]. Since it has been reported that psychological/emotional IPV precedes other types of IPV, such as physical or sexual [39,40], and this is the most frequent type of IPV in Peru, awareness about this type of violence must be raised even during courtship [41,42].

Although no association was found between women’s autonomy and sexual or physical IPV, this type of violence is not uncommon in Peru or in other LAC countries [31]. Although the prevalence of physical and/or sexual IPV, in general, has shown a downward trend in LAC in recent decades [31], this type of violence can have serious consequences for the health and the quality of life of the victimized woman. Characteristics such as financial autonomy and freedom of movement for women indicate a lower probability of suffering physical or sexual IPV [43], and thus, the promotion of autonomy in these aspects would be beneficial for reducing IPV. Paradoxically, in the literature, it is described that women who present sexual autonomy could have a greater probability of presenting IPV [44]. This might be explained by the fact that a woman with sexual autonomy could be seen as defiant by opposing coital relationships or fighting for her rights with her partner, who might react violently against her [44]. For this reason, programs for the prevention and fight against IPV must comprehensively address all types of IPV by promoting women’s autonomy and respect for them by their partners within the framework of a culture of respect and equality between people of different genders.

IPV not only affects the health of the woman but also the cognitive development of the children [45,46] and decreases the probability of the woman receiving institutional delivery care or adequate prenatal care leading to repercussions on maternal and child health outcomes [47,48]. IPV is a public health problem that deserves attention from decision-makers and health personnel in order to achieve early identification and implement preventive programs that improve the health status of women who are victims of this type of violence. Additionally, the COVID-19 pandemic context conferred a greater risk of violence, with some women who are victims of violence even having been forced to live with their aggressors (in Peru, the number of calls to call centers for complaints of family and sexual violence doubled during this period) [49]. Thus, there is an important need for the development of programs and policies aimed at identifying and caring for women whose vulnerability to IPV increased due to the pandemic.

Among the limitations of this study, causality could not be assessed due to the lack of temporality in the measurement of the study variables. Additionally, there could be memory bias and social desirability bias on the part of the interviewees, as well as errors in the recording of information by the interviewer. Despite these limitations, the source of information used (ENDES) is a population-based survey that allows the study of different development indicators or the health status of the Peruvian population, which is why it is useful for the study of the IPV and women’s autonomy. In addition, since the ENDES is a survey based on the DHS model, it has a widely supported methodology that allows comparison of the population’s health status over time and with respect to other countries in which surveys with the same methodology are also used.

## 5. Conclusions

IPV against women is frequent in the Peruvian population. Women with low autonomy have a higher probability of suffering IPV compared to those with high autonomy. This relationship was also specifically found among women experiencing the psychological/verbal type of IPV, but not in those describing physical or sexual IPV. Thus, the need for strategies and programs for the prevention of IPV against women to promote empowerment and increase the autonomy of women for decision-making in the different personal, family, and partner spheres is highlighted. Similarly, programs focused on women’s partners should be developed to promote respect and eliminate IPV. Additionally, given the complexity of the approaches to IPV and the influence that the woman’s level of autonomy may have, complementary studies using mixed approaches are necessary to delve into the study of the relationships evaluated in this study in the Peruvian population.

## Figures and Tables

**Table 1:** Variables that make up the autonomy of women.

Dimensions	Characteristics	Questions (ENDES Code)	Coding
Decision-making	Economy	Who has the last word in deciding what to do with the money the husband earns? (V743F)	It is coded as 1, when the woman had the last word in spending the husband’s money, and 0 when she did not.
		Who has the last word in making large household purchases? (V743B)	It was coded as 1, when the woman had the last word in making large purchases in the home, and 0 when she did not.
		Who has the last word in shopping for daily necessities? (V743C)	It was coded as 1, when the woman had the last word in making purchases for daily needs, and 0 when she did not.
	Health	Who has the last word in health care? (V743A)	It was coded as 1, when the woman had the last word in her health care, and 0 when she did not.
	Free movement	Who has the last word in visiting family or relatives? (V743D)	It was coded as 1, when the woman had the last word in visiting her family, and 0 when she did not.
Attitude towards violence	Justifies that she was beaten because she didn’t tell husband she was going out	Beaten wife justifies if she leaves without telling him (V744A)	It was coded as 1, when the woman justified the violence, and 0 when she did not.
	Justifies that she was beaten because she neglected the children	Beaten wife justifies if she neglects children (V744B)	
	Justifies that she was beaten because she argued with him	Beaten wife justifies if she argues with him (V744C)	
	Justifies that she was beaten because she did not have sex	Beaten wife justifies if she refuses to have sex with him (V744D)	
	Justifies that she was beaten because she burned the food	Beaten wife justifies if she burns food (V744E)	
Socio-economic aspects	Employment status in the last 12 months	Work in the last 12 months (V731)	It was coded as 1, when she worked in the last 12 months, and 0 when she did not.
	Head of household	Sex of the head of household (V151)	It was coded as 1, when the head of the household was a woman, and 0 when he was a man.
Socio-cultural aspects	Educational level	Highest level of education (V106)	It was coded as 0, when the woman had no education or only primary education; 1, when she studied secondary, and 2, when she had a higher education
	Access to television	Frequency with which you watch television (V159)	It was coded as 1, when the woman watched television at least once a week or every day, and 0, when she did not watch television or did so less than once a week.
	Access to radio	Frequency with which you listen to the radio (V158)	It was coded as 1, when the woman listened to the radio at least once a week or every day, and 0, when she did not listen to the radio or did so less than once a week.
	Access to newspaper	Frequency with which you read the newspaper or magazine (V157)	It was coded as 1, when the woman read the newspaper or magazine at least once a week or every day, and 0, when she did not read the newspaper or magazine or read it less than once a week.

ENDES: Demographic and Family Health Survey.

**Table 2:** Characteristics of Peruvian women aged 15 to 49 years included in the study.

Characteristic	Absolute Frequency (*n* = 18,621)	Weighted Proportion *
Age group of women		
35–49	3201	12.0
25–34	8197	37.7
15–24	7223	50.3
Ethnicity		
Non-native	16,690	93.4
Native	1931	6.6
Education level		
Higher	5815	35.0
Secondary	8275	43.2
No formal education/Primary	4531	21.8
Contraceptive use		
Yes	15,010	76.2
No	3611	23.8
Length of marriage		
0 to 9 years	8711	39.6
10 to 19 years	6734	37.7
20 years or more	3176	22.7
Number of children		
0 children	5638	35.7
1 to 3 children	12,644	63.0
4 to 7 children	339	1.3
Educational level of the couple		
Higher	6217	36.0
Secondary	9064	48.0
No formal education/Primary	3340	16.0
Alcohol use of partner		
No	3978	22.9
Yes	14,643	77.1
Place of residence		
Urban	12,553	76.9
Rural	6068	23.1
Wealth quintile		
Q5 (richest)	1858	16.8
Q4	2628	18.2
Q3	3607	21.2
Q2	5055	22.7
Q1 (poorest)	5473	21.1
Natural region		
Coast	7491	59.1
Highlands	6421	25.8
Jungle	4709	15.2

* The weighting factor and sample specifications of the ENDES 2019 were included.

**Table 3:** Characteristics of Peruvian women aged 15 to 49 years included in the study according to types of violence.

Characteristic	Intimate Partner Violence % (95% CI) *	*p*-Value **	Psychological/Verbal Violence % (95% CI) *	*p*-Value **	Physical Violence % (95% CI) *	*p*-Value **	Sexual Violence % (95% CI) *	*p*-Value **
Overall prevalence	40.1 (38.7–41.5)		38.8 (37.4–40.2)		8.8 (8.0–9.5)		2.3 (2.0–2.8)	
Age group of women								
35–49	48.1 (44.8–51.3)	<0.001	46.7 (43.4–50.0)	<0.001	12.4 (10.4–14.8)	<0.001	1.8 (1.3–2.5)	0.415
25–34	39.4 (37.3–41.5)		37.8 (35.8–39.9)		9.7 (8.6–10.9)		2.5 (1.9–3.3)	
15–24	38.7 (36.6–40.8)		37.6 (35.5–39.7)		7.2 (6.2–8.3)		2.3 (1.9–2.9)	
Ethnicity								
Non-native	39.9 (38.4–41.4)	0.195	38.6 (37.1–40.1)	0.165	8.7 (7.9–9.5)	0.190	2.3 (1.9–2.7)	0.008
Native	42.5 (39.0–46.0)		41.3 (37.9–44.8)		9.9 (8.3–11.7)		3.7 (2.7–5.0)	
Education level								
Higher	35.4 (32.9–38.0)	<0.001	33.9 (31.4–36.4)	<0.001	7.3 (6.0–8.7)	0.008	1.5 (1.0–2.3)	<0.001
Secondary	44.1 (41.9–46.3)		42.9 (40.7–45.1)		9.9 (8.8–11.1)		2.4 (1.9–3.0)	
No formal education/Primary	39.6 (37.2–42.1)		38.4 (36.0–40.9)		9.0 (7.8–10.3)		3.6 (2.9–4.5)	
Contraceptive use								
Yes	39.6 (38.0–41.2)	0.254	38.3 (36.8–39.9)	0.285	8.9 (8.1–9.8)	0.587	2.4 (2.0–2.8)	0.850
No	41.6 (38.5–44.9)		40.2 (37.1–43.5)		8.4 (7.0–10.1)		2.3 (1.5–3.4)	
Length of marriage								
0 to 9 years	39.1 (37.0–41.3)	0.298	37.8 (35.8–40.0)	0.155	9.2 (8.2–10.3)	0.243	1.7 (1.2–2.3)	0.016
10 to 19 years	39.8 (37.6–42.2)		38.2 (36.0–40.5)		9.0 (7.7–10.4)		2.7 (2.1–3.4)	
20 years or more	42.1 (38.9–45.3)		41.3 (38.2–44.5)		7.6 (6.3–9.1)		3.0 (2.2–4.0)	
Number of children								
0 children	38.3 (35.8–40.8)	0.126	37.3 (34.8–39.8)	0.219	7.8 (6.7–9.1)	0.116	1.8 (1.3–2.6)	0.070
1 to 3 children	41.1 (39.4–42.8)		39.6 (37.9–41.4)		9.2 (8.3–10.2)		2.6 (2.2–3.1)	
4 to 7 children	39.5 (30.7–49.0)		38.4 (29.7–47.9)		12.5 (6.7–22.2)		3.0 (1.6–5.8)	
Educational level of the couple								
Higher	35.8 (33.3–38.3)	<0.001	34.3 (31.9–36.8)	<0.001	7.4 (6.2–8.8)	0.015	1.3 (0.9–2.1)	<0.001
Secondary	43.2 (41.2–45.2)		41.9 (39.9–43.9)		9.3 (8.3–10.4)		2.6 (2.1–3.2)	
No formal education/Primary	40.4 (37.8–43.0)		39.5 (36.9–42.1)		10.1 (8.5–11.9)		3.8 (2.9–5.0)	
Alcohol use of partner								
No	35.8 (32.9–38.9)	0.001	35.2 (32.2–38.2)	0.006	6.5 (5.2–8.1)	0.003	1.3 (0.9–2.0)	0.002
Yes	41.3 (39.8–42.9)		39.8 (38.3–41.4)		9.4 (8.6–10.3)		2.7 (2.2–3.2)	
Place of residence								
Urban	40.3 (38.6–42.1)	0.384	39.1 (37.4–40.9)	0.268	8.5 (7.6–9.5)	0.106	2.0 (1.6–2.5)	<0.001
Rural	39.2 (37.4–41.0)		37.7 (35.9–39.5)		9.6 (8.7–10.6)		3.5 (2.9–4.2)	
Wealth quintile								
Q5 (richest)	32.8 (28.9–37.0)	<0.001	31.1 (27.3–35.2)	<0.001	5.6 (3.8–8.2)	<0.001	0.8 (0.3–1.9)	0.001
Q4	40.7 (37.0–44.4)		39.9 (36.3–43.6)		7.0 (5.5–8.9)		1.5 (0.8–2.8)	
Q3	41.8 (38.5–45.1)		40.4 (37.2–43.7)		8.9 (7.4–10.6)		2.7 (1.8–4.0)	
Q2	43.5 (41.0–46.0)		42.5 (40.0–45.0)		11.6 (10.1–13.4)		2.9 (2.3–3.6)	
Q1 (poorest)	39.9 (37.9–41.8)		38.3 (36.4–40.3)		9.5 (8.5–10.7)		3.4 (2.8–4.2)	
Natural region								
Coast	39.0 (36.8–41.2)	0.001	37.8 (35.7–40.0)	0.001	7.9 (6.9–9.1)	0.011	1.9 (1.4–2.5)	0.006
Highlands	43.7 (41.8–45.7)		42.5 (40.6–44.4)		9.9 (8.9–11.1)		2.9 (2.4–3.6)	
Jungle	38.0 (35.9–40.2)		36.2 (34.1–38.3)		9.9 (8.7–11.4)		3.2 (2.5–4.1)	

95% CI: 95% Confidence Interval. * The weighting factor and sample specifications of the ENDES 2019 were included. ** The *p*-value was calculated using the Chi-square test.

**Table 4:** Characteristics of Peruvian women aged 15 to 49 years included in the study according to level of autonomy.

Characteristic	High Autonomy % (95% CI) *	Moderate Autonomy % (95% CI) *	Low Autonomy % (95% CI) *	*p*-Value **
Overall prevalence	18.8 (17.7–20.0)	39.2 (37.8–40.6)	42.0 (40.6–43.4)	
Age group of women				
35–49	12.8 (10.9–14.9)	39.6 (36.6–42.8)	47.6 (44.5–50.7)	<0.001
25–34	19.6 (17.9–21.4)	42.4 (40.4–44.4)	38.0 (36.0–40.1)	
15–24	19.7 (17.9–21.5)	36.7 (34.6–38.9)	43.6 (41.5–45.8)	
Ethnicity				
Non-native	19.6 (18.4–20.9)	40.5 (39.1–42.0)	39.9 (38.4–41.4)	<0.001
Native	7.4 (4.8–11.4)	20.4 (17.5–23.7)	72.1 (68.1–75.8)	
Education level				
Higher	43.0 (40.3–45.6)	45.0 (42.4–47.6)	12.1 (10.3–14.1)	<0.001
Secondary	8.4 (7.2–9.8)	47.7 (45.6–49.9)	43.8 (41.9–45.8)	
No formal education/Primary	0.6 (0.4–0.9)	13.0 (11.2–15.1)	86.4 (84.3–88.2)	
Contraceptive use				
Yes	18.2 (16.9–19.5)	40.2 (38.7–41.7)	41.7 (40.1–43.3)	0.069
No	20.8 (18.1–23.8)	36.2 (33.0–39.4)	43.1 (39.9–46.3)	
Length of marriage				
0 to 9 years	23.8 (22.0–25.8)	41.9 (39.9–43.9)	34.3 (32.3–36.3)	<0.001
10 to 19 years	18.0 (16.2–20.1)	40.1 (37.9–42.5)	41.8 (39.7–44.0)	
20 years or more	11.3 (9.2–13.9)	32.9 (29.8–36.2)	55.7 (52.3–59.1)	
Number of children				
0 children	22.1 (20.1–24.3)	40.8 (38.2–43.4)	37.1 (34.7–39.5)	<0.001
1 to 3 children	17.3 (15.9–18.8)	38.6 (36.9–40.3)	44.1 (42.5–45.8)	
4 to 7 children	1.9 (0.7–5.5)	25.2 (18.2–33.7)	72.9 (64.2–80.1)	
Educational level of the couple				
Higher	34.4 (32.0–36.9)	44.8 (42.4–47.3)	20.8 (18.8–22.9)	<0.001
Secondary	12.3 (10.9–13.8)	41.4 (39.4–43.4)	46.4 (44.3–48.4)	
No formal education/Primary	3.3 (2.3–4.6)	20.1 (17.7–22.7)	76.6 (73.9–79.1)	
Alcohol use of partner				
No	19.2 (16.5–22.3)	35.0 (31.7–38.4)	45.8 (42.6–49.0)	0.012
Yes	18.7 (17.5–20.0)	40.5 (38.9–42.0)	40.9 (39.3–42.4)	
Place of residence				
Urban	23.1 (21.7–24.6)	44.0 (42.2–45.7)	33.0 (31.3–34.7)	<0.001
Rural	4.5 (3.8–5.3)	23.3 (21.7–25.0)	72.2 (70.3–73.9)	
Wealth quintile				
Q5 (richest)	40.0 (35.8–44.2)	45.2 (41.0–49.4)	14.9 (12.0–18.3)	<0.001
Q4	27.5 (24.4–30.9)	44.4 (40.7–48.2)	28.0 (24.3–32.1)	
Q3	18.7 (16.5–21.1)	47.6 (44.4–50.8)	33.7 (30.6–36.9)	
Q2	11.0 (9.5–12.7)	41.4 (38.9–43.9)	47.6 (45.2–50.1)	
Q1 (poorest)	2.9 (2.4–3.6)	19.2 (17.7–20.8)	77.9 (76.1–79.5)	
Natural region				
Coast	22.2 (20.4–24.1)	44.1 (42.0–46.3)	33.7 (31.6–35.9)	<0.001
Highlands	15.0 (13.6–16.5)	32.8 (31.0–34.8)	52.2 (50.0–54.3)	
Jungle	12.1 (10.8–13.5)	30.8 (28.6–33.1)	57.1 (54.8–59.5)	

95% CI: 95% Confidence Interval. * The weighting factor and sample specifications of the ENDES 2019 were included. ** The *p*-value was calculated using the Chi-square test.

**Table 5:** Association between women’s autonomy and intimate partner violence in the last 12 months.

	Total Violence		Psycho Verbal		Physical		Sexual	
Characteristic	Crude Model	Model 1 *	Crude Model	Model 2 *	Crude Model	Model 3 *	Crude Model	Model 4 *
	PR (95% CI)	aPR (95% CI)	PR (95% CI)	aPR (95% CI)	PR (95% CI)	aPR (95% CI)	PR (95% CI)	aPR (95% CI)
Women’s autonomy								
High	Reference	Reference	Reference	Reference	Reference	Reference	Reference	Reference
Moderate	**1.13 (1.01–1.27)**	1.05 (0.94–1.18)	**1.16 (1.03–1.30)**	1.07 (0.95–1.20)	1.29 (0.98–1.72)	1.16 (0.86–1.55)	1.06 (0.54–2.11)	0.80 (0.38–1.66)
Low	**1.22 (1.09–1.36)**	**1.15 (1.01–1.31)**	**1.24 (1.10–1.39)**	**1.15 (1.01–1.31)**	**1.54 (1.17–2.03)**	1.39 (0.98–1.97)	1.88 (0.99–3.59)	1.09 (0.52–2.30)

PR: prevalence ratio, aPR: adjusted prevalence ratio. * Adjusted for woman’s age, woman’s ethnic self-identification, current contraceptive use, relationship duration, number of children, partner’s age, partner’s education level, partner’s alcohol intake, wealth quintile, place of residence and natural region of residence. Figures in bold had a *p* < 0.05.

## Data Availability

Publicly available datasets were analyzed in this study. These data can be found here: http://iinei.inei.gob.pe/microdatos/, accessed on 3 July 2021.

## References

[1] Krug E.G., Mercy J.A., Dahlberg L.L., Zwi A.B. (2002). The world report on violence and health. Lancet.

[2] Horton R. (2015). Offline: Gender equality—The neglected SDG for health. Lancet.

[3] Sardinha L., Maheu-Giroux M., Stöckl H., Meyer S.R., García-Moreno C. (2022). Global, regional, and national prevalence estimates of physical or sexual, or both, intimate partner violence against women in 2018. Lancet.

[4] Institute for Health Metrics and Evaluation Intimate Partner Violence—Level 2 Risk. http://www.healthdata.org/results/gbd_summaries/2019/intimate-partner-violence-level-2-risk.

[5] Schraiber L.B., D’Oliveira A.F.P.L., França-Junior I., Diniz S., Portella A.P., Ludermir A.B., Valença O., Couto M.T. (2007). Prevalência da violência contra a mulher por parceiro íntimo em regiões do Brasil. Rev. Saúde Pública.

[6] Urke H.B., Mittelmark M.B. (2015). Associations between intimate partner violence, childcare practices and infant health: Findings from Demographic and Health Surveys in Bolivia, Colombia and Peru. BMC Public Health.

[7] World Health Organization (2013). Global and Regional Estimates of Violence against Women: Prevalence and Health Effects of Intimate Partner Violence and Non-Partner Sexual Violence.

[8] Instituto Nacional de Estadística e Informática Perú (2020). Encuesta Demográfica y de Salud Familiar-ENDES 2019. https://www.datosabiertos.gob.pe/dataset/encuesta-nacional-demograf%C3%ADa-y-salud-familiar-endes-2019-instituto-nacional-de-estad%C3%ADstica-e.

[9] Rondon M. (2003). From Marianism to terrorism: The many faces of violence against women in Latin America. Arch. Women’s Ment. Health.

[10] Bucheli M., Rossi M. (2019). Attitudes Toward Intimate Partner Violence Against Women in Latin America and the Caribbean. SAGE Open.

[11] Benavides M., León J., Etesse M., Espezúa L., Stuart J. (2019). Exploring the association between segregation and physical intimate partner violence in Lima, Peru: The mediating role of gender norms and social capital. SSM-Popul. Health.

[12] Guedes R.N., Da Fonseca R.M.G.S. (2011). Autonomy as a structural need to face gender violence. Rev. Esc. Enferm. USP.

[13] Bengesai A.V., Khan H.T.A. (2020). Female autonomy and intimate partner violence: Findings from the Zimbabwe demographic and health survey, 2015. Cult. Health Sex..

[14] Tenkorang E. (2018). Women’s Autonomy and Intimate Partner Violence in Ghana. Int. Perspect. Sex. Reprod. Health.

[15] Gram L., Morrison J., Skordis-Worrall J. (2019). Organising Concepts of ‘Women’s Empowerment’ for Measurement: A Typology. Soc. Indic. Res..

[16] Mackey A., Petrucka P. (2021). Technology as the key to women’s empowerment: A scoping review. BMC Women’s Health.

[17] Malwade Basu A., Brij Koolwal G. (2005). Two Concepts of Female Empowerment: Some Leads from DHS Data on Womens Status and Reproductive Health. A Focus on Gender Collected Papers on Gender Using DHS Data.

[18] Instituto Nacional de Estadística e Informática Ficha Técnica. https://proyectos.inei.gob.pe/endes/2018/documentos_2018/FICHA_TECNICA_ENDES_2018.pdf.

[19] Mganga A.E., Renju J., Todd J., Mahande M.J., Vyas S. (2021). Development of a women’s empowerment index for Tanzania from the demographic and health surveys of 2004–05, 2010, and 2015–16. Emerg. Themes Epidemiol..

[20] Rettig E.M., Fick S.E., Hijmans R.J. (2020). The Female Empowerment Index (FEMI): Spatial and temporal variation in women’s empowerment in Nigeria. Heliyon.

[21] Asaolu I.O., Alaofè H., Gunn J.K.L., Adu A.K., Monroy A.J., Ehiri J.E., Hayden M.H., Ernst K.C. (2018). Measuring Women’s Empowerment in Sub-Saharan Africa: Exploratory and Confirmatory Factor Analyses of the Demographic and Health Surveys. Front. Psychol..

[22] Wado Y.D. (2018). Women’s autonomy and reproductive health-care-seeking behavior in Ethiopia. Women Health.

[23] Murshid N.S., Critelli F.M. (2020). Empowerment and Intimate Partner Violence in Pakistan: Results from a Nationally Representative Survey. J. Interpers. Violence.

[24] Ram R., Kumar M., Kumari N. (2022). Association between women’s autonomy and unintended pregnancy in India. Clin. Epidemiol. Glob. Health.

[25] Zegenhagen S., Ranganathan M., Buller A.M. (2019). Household decision-making and its association with intimate partner violence: Examining differences in men’s and women’s perceptions in Uganda. SSM-Popul. Health.

[26] Ahinkorah B.O., Dickson K.S., Seidu A.-A. (2018). Women decision-making capacity and intimate partner violence among women in sub-Saharan Africa. Arch. Public Health.

[27] Gautam S., Jeong H.-S. (2019). The Role of Women’s Autonomy and Experience of Intimate Partner Violence as a Predictor of Maternal Healthcare Service Utilization in Nepal. Int. J. Environ. Res. Public Health.

[28] Instituto Nacional de Estadística e Informática ENDES Documentos Metodológicos. https://proyectos.inei.gob.pe/endes/documentos.asp.

[29] FRA (European Union Agency for Fundamental Rights) Violence against Women: An EU-Wide Survey. Main Results Report..

[30] World Health Organization Intimate Partner Violence. https://apps.who.int/iris/bitstream/handle/10665/77432/WHO_RHR_12.36_eng.pdf;sequence=1.

[31] Bott S., Guedes A., Ruiz-Celis A.P., Mendoza J.A. (2019). Intimate partner violence in the Americas: A systematic review and reanalysis of national prevalence estimates. Rev. Panam. Salud Publica Pan Am. J. Public Health.

[32] Ministerio de la Mujer y Poblaciones Vulnerables Violencia Basada En Género Marco Conceptual Para Las Políticas Públicas y La Acción Del Estado. https://www.mimp.gob.pe/files/direcciones/dgcvg/MIMP-violencia-basada_en_genero.pdf.

[33] Ministerio de la Mujer y Poblaciones Vulnerables ESTRATEGIA NACIONAL DE PREVENCIÓN DE LA VIOLENCIA DE GÉNERO CONTRA LAS MUJERES “MUJERES LIBRES DE VIOLENCIA”. https://observatorioviolencia.pe/wp-content/uploads/2021/07/Estrategia-Mujeres-libres-de-violencia.pdf.

[34] World Health Organization Understanding and Addressing Violence against Women: Intimate Partner Violence. https://apps.who.int/iris/handle/10665/77432.

[35] World Health Organization Global and Regional Estimates of Violence against Women. https://www.who.int/publications/i/item/9789241564625.

[36] Mavisakalyan A., Rammohan A. (2020). Female autonomy in household decision-making and intimate partner violence: Evidence from Pakistan. Rev. Econ. Househ..

[37] Forty J. (2021). Do women with autonomy in the household experience less intimate partner violence in Malawi? Evidence from the 2015–16 Demographic and Health Survey. J. Biosoc. Sci..

[38] Svec J., Andic T. (2018). Cooperative Decision-Making and Intimate Partner Violence in Peru. Popul. Dev. Rev..

[39] Salis K.L., Salwen J., O’Leary K.D. (2014). The Predictive Utility of Psychological Aggression for Intimate Partner Violence. Partn. Abus..

[40] Schumacher J.A., Leonard K.E. (2005). Husbands’ and Wives’ Marital Adjustment, Verbal Aggression, and Physical Aggression as Longitudinal Predictors of Physical Aggression in Early Marriage. J. Consult. Clin. Psychol..

[41] Valdivia-Peralta M., Fonseca-Pedrero E., Bravo L.G., Piñeiro M.P. (2019). Invisibilización de la violencia en el noviazgo en Chile: Evidencia desde la investigación empírica. Perfiles Latinoam..

[42] Anacona C.A.R. (2008). Prevalencia, factores de riesgo y problemáticas asociadas con la violencia en el noviazgo: Una revisión de la literatura. Av. Psicol. Latinoam..

[43] Sabarwal S., Santhya K.G., Jejeebhoy S.J. (2014). Women’s Autonomy and Experience of Physical Violence Within Marriage in Rural India: Evidence from a Prospective Study. J. Interpers. Viol..

[44] Aboagye R.G., Dadzie L.K., Arthur-Holmes F., Okyere J., Agbaglo E., Ahinkorah B.O., Seidu A.-A. (2022). Intimate partner violence against married and cohabiting women in sub-Saharan Africa: Does sexual autonomy matter?. Reprod. Health.

[45] Howell K.H., Barnes S.E., Miller L.E., Graham-Bermann S.A. (2016). Developmental variations in the impact of intimate partner violence exposure during childhood. J. Inj. Viol. Res..

[46] Mueller I., Tronick E. (2019). Early Life Exposure to Violence: Developmental Consequences on Brain and Behavior. Front. Behav. Neurosci..

[47] Ward C.L., Harlow S. (2021). RESPecT and intimate partner violence: A cross-sectional study using DHS data in Kenya. BMJ Open.

[48] Sripad P., Hossain S., Ndwiga C., Warren C. (2019). Autonomy, Intimate Partner Violence, and Maternal Health-Seeking Behavior: Findings from Mixed-Methods Analysis in Bangladesh.

[49] Ministerio de la Mujer y Poblaciones Vulnerables. Línea 100 del MIMP incrementó en 97% las atenciones de llamadas durante el 2020. https://www.gob.pe/institucion/mimp/noticias/325922-linea-100-del-mimp-incremento-en-97-las-atenciones-de-llamadas-durante-el-2020.

